# Application of Colloidal Dispersions of Bioshell Calcium Oxide (BiSCaO) for Disinfection

**DOI:** 10.3390/polym11121991

**Published:** 2019-12-02

**Authors:** Yoko Sato, Heisuke Ohata, Akinori Inoue, Masayuki Ishihara, Shingo Nakamura, Koichi Fukuda, Tomohiro Takayama, Kaoru Murakami, Sumiyo Hiruma, Hidetaka Yokoe

**Affiliations:** 1 Division of Biomedical Engineering, Research Institute, National Defense Medical College, 3-2 Namiki, Tokorazawa, Saitama 359-8513, Japan; ysato@ndmc.ac.jp (Y.S.); res331@ndmc.ac.jp (H.O.); res011@ndmc.ac.jp (A.I.); snaka@ndmc.ac.jp (S.N.); res309@ndmc.ac.jp (K.F.); res337@ndmc.ac.jp (S.H.); 2 Department of Oral and Maxillofacial Surgery, National Defense Medical College, 3-2 Namiki, Tokorozawa, Saitama 359-8513, Japan; taka01@ndmc.ac.jp (T.T.); murakami@ndmc.ac.jp (K.M.); yokoe@ndmc.ac.jp (H.Y.)

**Keywords:** bioshell powder, calcium oxides, colloidal dispersion, microbicidal activity, disinfection, flocculation/precipitation

## Abstract

Bioshell calcium oxide (BiSCaO) is a scallop-shell powder heated at a high temperature. BiSCaO is composed mainly of calcium oxide and exhibits broad microbicidal properties. The aim of this study is to evaluate the disinfection and decontamination abilities of BiSCaO colloidal dispersions with that of commercially available bioshell calcium hydroxide (BiSCa(OH)_2_) following the formation of flocculants/precipitates under strongly alkaline conditions (pH 11.5–12.2). Various concentrations of BiSCaO and BiSCa(OH)_2_ colloidal dispersions were prepared by mixing with Na-polyPO_4_ (PP) and Na-triPO_4_ (TP) as flocculating agents. The microbicidal activities, and the degree of flocculation/precipitation of trypan blue, albumin, chondroitin sulfate, heparin, non-anticoagulant heparin carrying polystyrene (NAC-HCPS), and low-molecular-weight heparin/protamine nanoparticles (LMWH/P NPs) were dependent on the pH, the average particle diameter, and the concentration of BiSCaO or BiSCa(OH)_2_ and of the phosphate compound. BiSCaO (average particle diameter: 6 μm) colloidal dispersions (0.2 wt.%) containing 0.15 wt.% PP or TP exhibited substantially stronger microbicidal activity and flocculation/precipitation under strongly alkaline conditions. These results suggest that BiSCaO colloidal dispersions together with phosphate compounds have practical applicability for disinfection.

## 1. Introduction

Calcium oxide produced from limestone (LiMCaO) is generally readily available and is an important inorganic compound used in various industries as, for example, an adsorbent, toxic-waste remediation agent, and an alkalization agent. However, LiMCaO contains harmful impurities and has a dangerously high heat of hydration [1,2]. On the other hand, scallop shells are readily available safe sources of calcium oxide (CaO) and Ca(OH)_2_ and are used as a food additive, which have moderate heats of hydration following appropriate processing. Most scallop shells are originally considered industrial waste and accumulate on the shores of harvesting districts in Japan, causing serious problems such as offensive odors and soil pollution due to harmful materials leaching from the shells if left as they are [1,3]. The harmful materials in scallop shells could be removed by heating at a high temperature, and by grinding and sieving. Therefore, an increasing amount of attention has been paid to practical applications of CaO and Ca(OH)_2_ derived from scallop shells.

The main component of scallop shells is calcium carbonate (CaCO_3_), which is converted to CaO when heated at over 800 °C. Heated scallop shell powder is composed mainly of CaO, which we called bioshell calcium oxide (BiSCaO), a material well known to exhibit strong antimicrobial activity [4]. For example, BiSCaO shows broad antimicrobial action against the avian influenza virus [5], bacteria [3,4], heat-resistant bacterial spores [3,6], fungi [7], and biofilms [8,9,10]. In addition, BiSCaO is used as an additive to prolong the shelf life of food products [10]. CaO is easily converted to Ca(OH)_2_ by hydration with water and air moisture. CaO hydration generates a strong base, considered the primary mechanism for the antimicrobial action of BiSCaO. For example, the antimicrobial activity of the BiSCaO reaction towards both total viable cells (TC) and coliform bacteria (CF) is higher than that of Ca(OH)_2_ or NaOH solutions at the same pH [11,12]. Furthermore, BiSCaO can reduce formaldehyde [13]. Slurries of heated scallop shell powder (particle diameter range: 60–900 nm) are prepared by grinding shells heated to over 1100 °C in a wet bead-grinding mill [4] and suspending the powder in sterile saline. The main component of this shell powder slurry is Ca(OH)_2_ generated by the hydration of CaO. Similarly, most commercially available heated shell powder products (BiSCa(OH)_2_) used as food additives are composed mainly of Ca(OH)_2_. In this study, we purchased BiSCaO composed of more than 99% CaO with a particle diameter of 6 µm from Plus Lab Co. Ltd., Kanagawa, Japan [14]. 

Both BiSCaO and BiSCa(OH)_2_ are poorly water-soluble under strongly alkaline conditions (pH > 11.5). Consequently, water suspensions of high concentrations of BiSCaO and BiSCa(OH)_2_ can result in significant loss and can plug spray nozzles due to precipitation [14,15]. We previously reported preventing the precipitation of a BiSCaO dispersion by adding phosphate compounds such as Na_3_PO_4_, Na_2_HPO_4_, or NaH_2_PO_4_. These dispersions showed higher deodorization and microbicidal activities than BiSCa(OH)_2_, which is mainly composed of Ca(OH)_2_. BiSCaO, but not BiSCa(OH)_2_, can reduce NO_2_^−^ and NO_3_^−^. These activities of BiSCaO might result from the high pH (≥11.5) caused by CaO hydration and reductive activity resulting in the generation of active radical species [11,14].

Treatment of heated lime composed of mainly CaO and/or Ca(OH)_2_ reduces the number of microorganisms by flocculation in sedimentation or flotation processes and, at the same time, the hydroxide alkalinity has a strong microbicidal effect (≥11.5) [16]. In fact, alkaline flocculation/precipitation with calcium phosphate is a technically feasible and low-cost method for removing negatively charged organic compounds or nanoparticles including organic matter from bacteria such as endotoxin as well as microbicidal activity. For example, model wastewater components interfere efficiently with the alkaline flocculation/precipitation induced by calcium phosphate precipitates [17] and this flocculation was unaffected by inorganic nitrogen, sodium alginate, and salinity whereas bovine serum albumin and organic matter from *Escherichia coli* strongly decreased with the alkaline flocculation/precipitation with calcium phosphate [16].

In this study, we established a method to prevent the precipitation of a 0.2 wt.% BiSCaO and BiSCa(OH)_2_ colloidal dispersion prepared by mixing with Na-polyPO_4_ (PP) or Na-triPO_4_ (TP). Specifically, we established conditions for optimally generating 0.2 wt.% BiSCaO and BiSCa(OH)_2_ colloidal dispersions by the addition of 0.15 wt.% PP and adjusting the pH to 11.5–12.2. We then studied the resulting BiSCaO colloidal dispersions for their disinfecting effects towards normal bacterial flora (total viable cells (TC) and coliform bacteria (CF)). Subsequently, we studied on the abilities of these BiSCaO colloidal dispersions to flocculate and precipitate trypan blue, albumin, chondroitin sulfate, heparin, non-anticoagulant heparin carrying polystyrene (NAC-HCPS) [18,19], and low-molecular-weight heparin/protamine nanoparticles (LMWH/P NPs) as negatively charged organic compounds or nanoparticles [20,21] which, to compare to those of BiSCa(OH)_2_ colloidal dispersions.

## 2. Materials and Methods

### 2.1. BiSCaO and BiSCa(OH)_2_ Powders and Chemicals

Scallop shell powders were heated at 1450 °C for 4 h, then ground using a dry super grinder (Nano Jetmizer NJ-300-D, Aishin Nano Technologies Co. Ltd., Saitama, Japan), followed by cooling in a vacuum chamber. This provided BiSCaO with dry powder diameters of 3–9 (average 6 μm) and was purchased from Plus Lab Corp., Kanagawa, Japan. According to the manufacturer, the content of CaO in this BiSCaO preparation was 99.6%. BiSCa(OH)_2_ was obtained from Scallow, Kohkin Inst. Co. Ltd., Tochigi, Japan, had a dry powder diameter of 10–100 μm (average 46 μm), and the CaO and Ca(OH)_2_ concentrations were <5% and >90%, respectively. Sodium polyphosphate (Na-polyPO_4_; PP), sodium triphosphate (Na-triPO_4_; TP), 0.4 *w*/*v*% trypan blue solution, and 1 N HCl were purchased from FUJI FILM Wako Pure Chemical Corp., Osaka, Japan. Chondroitin sulfate and heparin were purchased from SEIKAGAKU Corp., Tokyo, Japan. Non-anticoagulant heparin carrying polystyrene (NAC-HCPS) [18,19] and low-molecular-weight heparin/protamine nanoparticles (LMWH/P NPs) [20,21] were prepared as previously described. 

### 2.2. BiSCaO and BiSCa(OH)_2_ Colloidal Dispersions with Na-polyPO_4_ (PP) or Na-triPO_4_ (TP)

BiSCaO (0.2 g) or BiSCa(OH)_2_ was added to 100 mL of pure water, followed by rotary mixing, to generate 0.2 wt.% suspensions, then either 0.05, 0.1, 0.15, 0.2, 0.25 or 0.3 wt.% PP or TP was added to 10 mL of each suspension. Various amounts of 1 N HCl were added to the 0.2 wt.% BiSCaO or BiSCa(OH)_2_ colloidal dispersion containing 0.15 wt.% of PP or TP to adjust pH, from strongly alkaline (>12) to weakly acidic (≈5). The pH values were measured with a pH meter (F-70, HORIBA Ltd., Kyoto, Japan). Each colloidal dispersion was evaluated on the form such as suspension with precipitates, dispersion, and colloidal dispersion with flocculants/precipitates after centrifugation at 500 rpm (50× *g*) for 5 min, and the degree of layer separation with flocculants/precipitates (white insoluble layer) to the total amount was calculated. The zeta potentials of BiSCaO and LiMCaO colloidal dispersions at various pH values were determined using an ELSZ-1000 particle analyzer (Otsuka Electronics Co. Ltd., Osaka, Japan).

### 2.3. Scanning Electron Microscopy (SEM) and Cryo-images of BiSCaO Dry Powder and BiSCaO Colloidal Dispersions with PP

Scanning electron microscopy (SEM) images of the dry powders were obtained by osmium metal coating using a neo-osmium coater (Neoc-STB; Meiwafosis Co., Ltd., Tokyo, Japan). The surface structure of each dry powder was observed from SEM images obtained using a field-resolved scanning electron microscope (JSM-6340F; JEOL Ltd. Tokyo, Japan). For cryo-SEM [22,23], a 0.2 wt.% BiSCaO colloidal dispersion containing 0.15 wt.% PP was frozen in liquid nitrogen, then knife-cut and observed using a JEOL JSM 7100F SEM (JEOL Ltd., Tokyo, Japan) under vacuum conditions at −90 °C. The accelerating voltage was 10 KV, and the detection signal was a backscattered electron image. 

### 2.4. Flocculation and Precipitation of Trypan Blue with BiSCaO and BiSCa(OH)_2_ Colloidal Dispersions

First, 0.05, 0.1, 0.15, 0.2, 0.25 or 0.3 wt.% of PP or PT was added to 10 mL of 0.2 wt.% BiSCaO or BiSCa(OH)_2_ suspensions and mixed well. Next, 100 μL of 0.4 wt./vol.% trypan blue solution was added to each colloidal dispersion and vortexed for 10 s. The generated flocculants/precipitates with trypan blue was separated by centrifugation (1000 rpm, 190× *g*) for 5 min.

Various amounts of 1 N HCl were added to the 0.2 wt.% BiSCaO or BiSCa(OH)_2_ colloidal dispersions (10 mL) containing 0.15 wt.% PP or PT to adjust the pH, from strongly alkaline (>12) to weakly acidic (≈5), then 100 μL of 0.4 wt./vol.% trypan blue solution was added to each colloidal dispersion and vortexed for 10 s. The generated flocculants/precipitates with trypan blue were separated by centrifugation (1000 rpm, 190× *g*) for 5 min. The samples were photographed and the optical densities of the supernatants at 620 nm were measured using a spectrophotometer (AE-450N, ERMA Inc., Tokyo, Japan).

### 2.5. Flocculation and Precipitations of Albumin with BiSCaO and BiSCa(OH)_2_ Colloidal Dispersions

PP or PT (0.1, 0.2, 0.3, 0.4, 0.5 or 0.6 wt.%) was added to 0.5 mL of 0.4 wt.% BiSCaO or BiSCa(OH)_2_ suspensions and mixed well. Next, 0.5 mL of 1 mg/mL (0.1 wt.%) albumin solution was added to each colloidal dispersion and vortexed for 10 sec. The generated flocculants/precipitates with albumin was separated by centrifugation (1000 rpm, 190× *g*) for 5 min. 

Various amounts of 1 N HCl were added to 0.5 mL of the 0.4 wt.% BiSCaO or BiSCa(OH)_2_ colloidal dispersions containing 0.3 wt.% PP and TP to adjust the pH, from strongly alkaline (>12) to weakly acidic (≈5), then 0.5 mL of 0.1 wt.% albumin was added to each colloidal dispersion and vortexed for 10 s. The generated flocculants/precipitates with albumin was separated by centrifugation (1000 rpm, 190× *g*) for 5 min. The optical densities of the supernatants at 562 nm were measured using a spectrophotometer.

### 2.6. Flocculation and Precipitation of Chondroitin Sulfate, Heparin, Non-Anticoagulant Heparin Carrying Polystyrene (NAC-HCPS) and Low-Molecular-Weight Heparin/Protamine Nanoparticles (LMWH/P NPs) with BiSCaO and BiSCa(OH)_2_ Colloidal Dispersions

Various amounts of 1 N HCl were added to 0.5 mL of 0.4 wt.% BiSCaO or BiSCa(OH)_2_ colloidal dispersions containing 75 wt.% PP compared with BiSCaO or BiSCa(OH)_2_ to adjust the pH, from strongly alkaline (>12) to weakly acidic (≈5), then 0.5 mL of 0.1 wt.% 0.1 mg/mL (0.01 wt.%) chondroitin sulfate, heparin, NAC-HCPS or LMWH/P NPs was added and each mixture was vortexed for 10 sec. Each generated flocculent/precipitate was separated by centrifugation (1000 rpm, 190× *g*) for 5 min. Chondroitin sulfate, heparin, and NAC-HCPS remaining in the supernatant were measured using a Blyscan Glycosaminoglycan Assay Kit (Wako Pure Chemical Industries, Ltd., Osaka, Japan) and the optical densities of the supernatants at 656 nm were measured using a spectrophotometer.

### 2.7. Microbicidal Efficacies of BiSCaO and BiSCa(OH)_2_ Colloidal Dispersions

Various amounts of 1 N HCl were added to 5 mL of 0.4 wt.% BiSCaO or BiSCa(OH)_2_ colloidal dispersions containing 75 wt.% PP compared with BiSCaO or BiSCa(OH)_2_ to adjust the pH from, strongly alkaline (>12) to weakly acidic (≈5). A suspension of normal bacterial flora (total viable cells (TC) and coliform bacteria (CF)) was prepared by incubating the leftover bath water with 10% Dulbecco’s Modified Eagle’s Medium (DMEM) at 37 °C for 24 h [11,24,25]. Each colloidal dispersion (5 mL) was added to 5 mL of the bacterial suspension, mixed well, and incubated at room temperature for 15 min, then the number of colony-forming units (CFU) per sample was determined by pouring 1 mL of each mixture gently into individual Petri-dishes containing pre-aliquoted portions of simple and easy dry medium for TC or CF (Nissui Pharmaceutical Co., Ltd., Tokyo, Japan) [11,24,25], then incubating the plates for 24 h in a 37 °C incubator (Alp Co., Ltd., Tokyo, Japan). 

## 3. Results

### 3.1. BiSCaO and BiSCa(OH)_2_ Colloidal Dispersions with Na-polyPO_4_ (PP) and Na-triPO_4_ (TP) 

**Figure 1:**
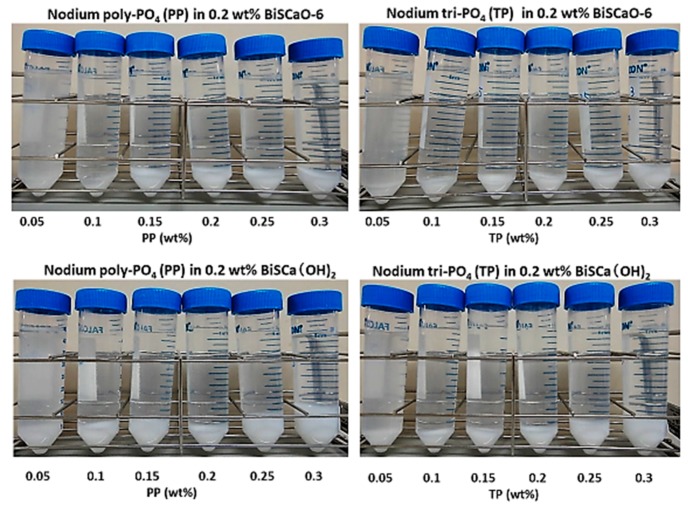
Various amounts of PP or PT were added to 10 mL of 0.2 wt.% BiSCaO or BiSCa(OH)_2_ suspensions and mixed well. The generated flocculants/precipitates layer was separated by centrifugation.

Various amounts of PP or TP as a flocculating agent were added to water suspensions of 0.2 wt.% BiSCaO or BiSCa(OH)_2_ adjusted to pH 12.2 with 1 N HCl. The results are shown in Figure 1 and Table 1. The addition of 0.1–0.3 wt.% PP or TP to water suspensions of 0.2 wt.% BiSCaO or BiSCa(OH)_2_ generated white colloidal dispersions which rapidly formed flocculants/precipitates layer (10–30 vol.%). The formed layer can be easily dispersed by several shakings. 

**Figure 2:**
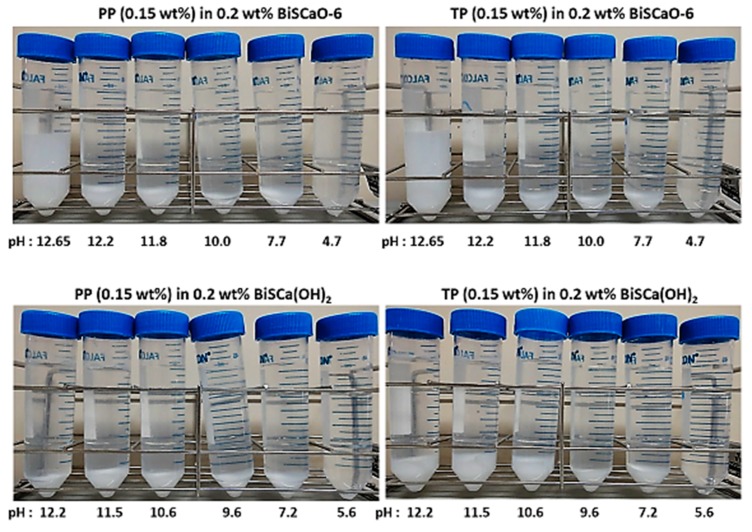
Various amounts of 1 N HCl were added to 0.2 wt.% BiSCaO or BiSCa(OH)_2_ colloidal dispersions containing 0.15 wt.% of PP or TP to adjust the pH. Each colloidal dispersion was evaluated for the degree of layer separation (flocculantes/precipitates) after centrifugation at 500 rpm (50× *g*) for 5 min.

PP or TP (0.15 wt.%) was added as a flocculating agent to water suspensions of 0.2 wt.% BiSCaO (pH 12.65) or BiSCa(OH)_2_ (pH 12.2) and the pH of the colloidal dispersion was adjusted by adding 1 N HCl to the indicated value (pH 4.5–12.2). The white colloidal dispersions rapidly formed easily dispersible flocculant layers (15–25 vol.%). In contrast, BiSCaO and BiSCa(OH)_2_ colloidal dispersions completely dissolved to provide a clear solution at pH = 4.7 and 5.6, respectively. BiSCaO suspension (0.2 wt.%) containing 0.15 wt.% PP or PT (pH = 12.65) remained as a dispersion and colloidal dispersion contained a flocculants/precipitates layer (Figure 2, Table 2). The zeta potentials of both the BiSCaO and BiSCa(OH)_2_ colloidal dispersions were over +10 mV at pH > 11.5 and −1–−5 mV at pH 7.2–12.65. (Table 2). 

### 3.2. Cryo-SEM Images of BiSCaO Dispersions Formed by Adding PP 

**Figure 3:**
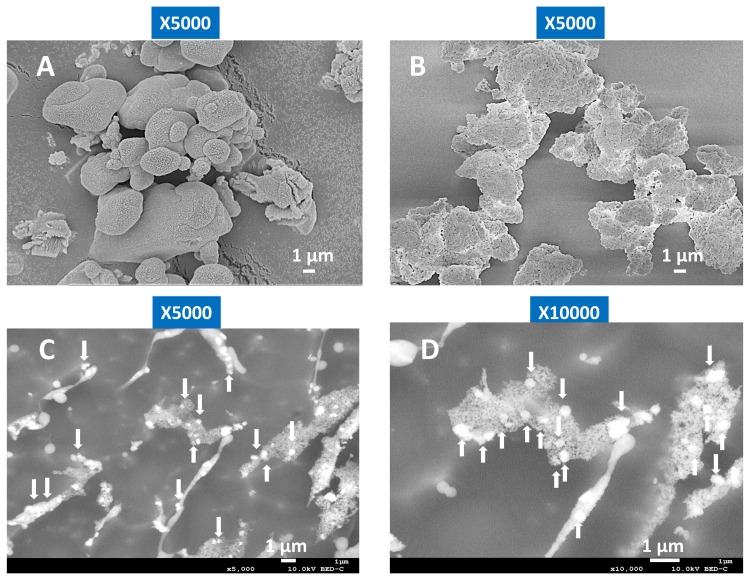
SEM images of BiSCaO dry powders and colloidal dispersions. The surface structure of a commercial dry powder of BiSCaO immediately after opening a sealed package, at 5,000× magnification (**A**) and BiSCaO-6 placed under high humidity at 37 °C for seven days, at 5,000× magnification (**B**). Images were obtained using a field-resolved scanning electron microscope. Cryo-SEM observations of BiSCaO colloidal dispersions (0.2 wt.% BiSCaO-6, 0.15 wt.% PP) two days after preparing dispersions**]** at 5,000× magnification (**C**) and 10,000× magnification (**D**). Arrows indicate BiSCaO-nanoparticles.

Commercial BiSCaO immediately after opening a sealed package is a polymorphic powder with a wrinkled surface structure (Figure 3A). In contrast, BiSCaO placed under high humidity at 37 °C for seven days exhibits a porous surface structure similar to nanoparticle aggregates (Figure 3B) that look like BiSCa(OH)_2_ powder just after opening a sealed package (data not shown). These observations suggested that surface hydration of BiSCaO particles may lead to the structural changes of CaO crystals and rift and pore formation, which may promote the production of nanoparticles from microparticles in the colloidal dispersion. Indeed, cryo-SEM (Figure 3C,D) indicate that CaO particles of nanoscale (150–300 nm) were surrounded by a cotton/fibrous lattice of colloidal calcium phosphate in BiSCaO colloidal dispersions. Colloidal calcium phosphate was generated in both BiSCaO and BiSCa(OH)_2_ colloidal dispersions whereas BiSCa(OH)_2_ nanoparticles were absent from BiSCa(OH)_2_ colloidal dispersions (data not shown). 

### 3.3. Flocculation/Precipitation of Trypan Blue with BiSCaO and BiSCa(OH)_2_ Colloidal Dispersions

**Figure 4:**
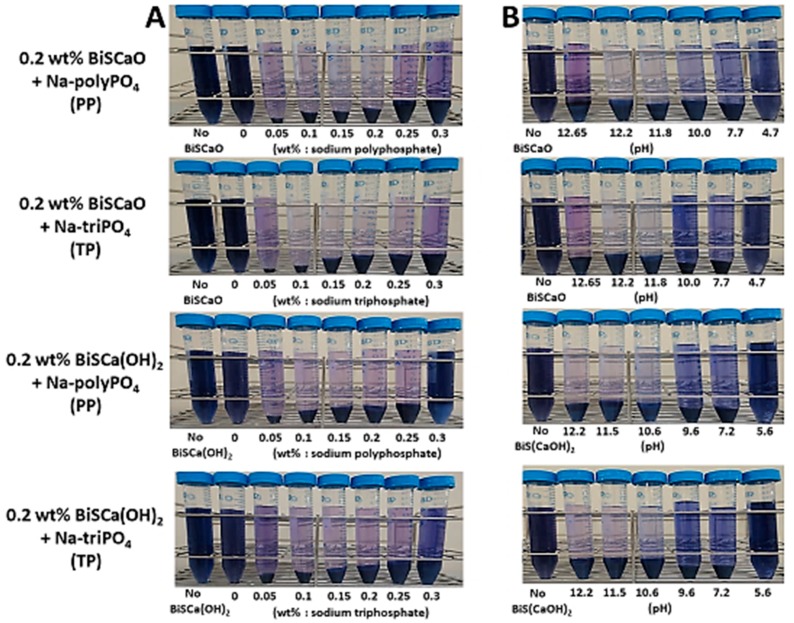
Various amounts of PP or PT were mixed with BiSCaO or BiSCa(OH)_2_ suspensions, then trypan blue was added (**A**). Various amounts of 1 N HCl were added to these BiSCaO or BiSCa(OH)_2_ colloidal dispersion to adjust the pH, then trypan blue was added (**B**). The photographs were taken after separation of the flocculants/precipitates containing trypan blue by centrifugation.

We determined the optimal amounts of PP and TP, and the optimal pH, required for the flocculation/precipitation of trypan blue in BiSCaO and BiSCa(OH)_2_ colloidal dispersions. Various amounts of PP or PT were mixed with BiSCaO or BiSCa(OH)_2_ suspensions (pH 12.2), then trypan blue was added. When 0.05–0.3 wt.% of either PP or TP was present in 0.2 wt.% BiSCaO or BiSCa(OH)_2_ colloidal dispersions (Figure 4A), more flocculants/precipitates of trypan blue was observed. A pH of 11.5–12.2 was optimum for the flocculation/precipitation of trypan blue in 0.2 wt.% BiSCaO or BiSCa(OH)_2_ colloidal dispersions containing 0.15 wt.% of PP and TP (Figure 4B). 

The optical densities of these supernatants were measured at 620 nm using a spectro-photometer (Table 3). We found that the amount of trypan blue in the supernatants of 0.2 wt.% BiSCaO or BiSCa(OH)_2_ colloidal dispersions containing 0.15 wt.% PP at pH 12.2 decreased by 98.6% and 92.4%, respectively. 

### 3.4. Flocculation/Precipitation of Albumin with BiSCaO and BiSCa(OH)_2_ Colloidal Dispersions

We determined the optimal amounts of PP and TP, and the optimal pH of BiSCaO or BiSCa(OH)_2_ colloidal dispersions, for the flocculation/precipitation of albumin using a method similar to that for trypan blue. Various amounts of PP or PT were mixed with 0.4 wt.% BiSCaO or BiSCa(OH)_2_ suspensions, then an equal volume of 0.1 wt.% albumin was added. The presence of 0.15 wt.% (final concentration) of either PP or TP in 0.2 wt.% final concentration of BiSCaO or BiSCa(OH)_2_ colloidal dispersions provided the highest flocculation/precipitation of albumin (Table 4) and the optimal pH was about 12.2 (Table 4). Taking these results together with those obtained using trypan blue, we chose 0.2 wt.% BiSCaO and BiSCa(OH)_2_ colloidal dispersions with 0.15 wt.% of PP as the optimal condition in further experiments. 

### 3.5. Flocculation/Precipitation of Chondroitin Sulfate, Heparin, NAC-HCPS, and LMWH/P NPs with BiSCaO and BiSCa(OH)_2_ Colloidal Dispersions

An equal volume of 0.1 mg/mL (0.01 wt.%) chondroitin sulfate, heparin, NAC-HCPS or LMWH/P NPs was added to a final concentration of 0.2 wt.% BiSCaO or BiSCa(OH)_2_ colloidal dispersion at the indicated pH, mixed, and incubated at room temperature for 15 min. The separation of two layers due to flocculation/precipitation was observed in all samples except under acidic conditions. Less chondroitin sulfate, heparin, NAC-HCPS, and LMWH/P NPs remained in the BiSCaO and BiSCa(OH)_2_ colloidal dispersion supernatants at pH 12.1 to 12.2 compared with colloidal dispersions under weakly acidic conditions (pH 5.3 to 5.7), and this difference was less pronounced under weakly basic and neutral conditions (pH 7 to 11) (Table 5). BiSCaO colloidal dispersion more effectively separated chondroitin sulfate, heparin, NAC-HCPS and LMWH/P NPs by flocculation/precipitation than did BiSCa(OH)_2_. The flocculation/precipitation of heparin was less pronounced than that of NAC-HCPS or LMWH/P NPs, even at pH 12.2. 

### 3.6. Antimicrobial Efficacy of BiSCaO and BiSCa(OH)_2_ Colloidal Dispersions

We investigated the antimicrobial efficacies of BiSCaO and BiSCa(OH)_2_ colloidal dispersions. A suspension of normal bacterial flora (total viable cells (TC) and coliform bacteria (CF)) was prepared by incubating leftover bath water with 10% DMEM at 37 °C for 24 h [24,25]. The TC and CF cell counts increased from 100 ± 45 and 65 ± 30 to 8.6 ± 1.5 (×10^6^) CFU/mL and 8.4 ± 1.8 (×10^6^) CFU/mL, respectively. 

**Figure 5:**
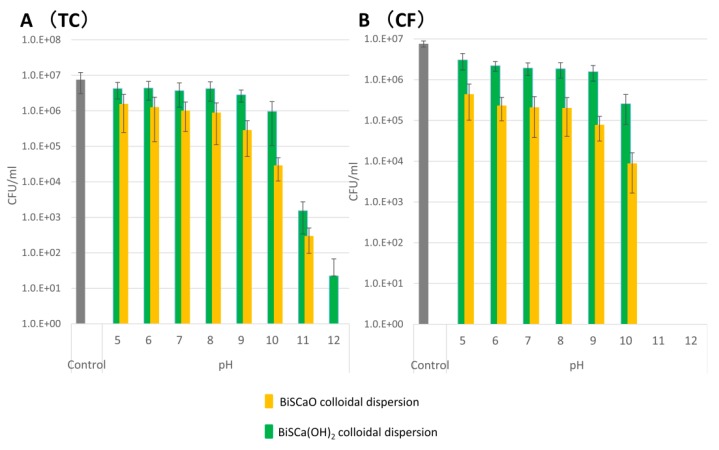
Various amounts of 1 N HCl were added to BiSCaO or BiSCa(OH)_2_ colloidal dispersions to adjust the pH, then an equal volume of bacterial suspension was added. The number of TC and CF colony forming units (CFUs) per 1 mL of each supernatant was determined.

An equal volume of the bacterial suspension and either BiSCaO colloidal dispersion or BiSCa(OH)_2_ colloidal dispersion at the indicated pH were mixed well and incubated at room temperature for 15 min. Separation of two layers due to flocculation/precipitation was observed in all samples, except under acidic conditions. The TC and CF cell counts in supernatants of the BiSCaO and BiSCa(OH)_2_ colloidal dispersions at pH ≥ 11 were much lower compared with samples at other pH values (pH ≤ 10), and the antimicrobial effect was more pronounced with BiSCaO colloidal dispersions than with BiSCa(OH)_2_ colloidal dispersions at each pH (pH 5–12). No TC and CF were observed following treatment with BiSCaO colloidal dispersion at pH 12 (Figure 5).

## 4. Discussion

Most commercially available heated-scallop shell powder products (BiSCa(OH)_2_) used as food additives are composed of Ca(OH)_2_. It is known that a scallop shell composed of calcium carbonate (CaCO_3_) is converted to calcium oxide (CaO) when heated above 800 °C. BiSCaO used in this study is prepared by heating shell powder at 1450 °C for 4 h to obtain over 99.6% CaO, grinding using a dry grinder, followed by cooling in a vacuum chamber and vacuum packing. The produced fine CaO powder, BiSCaO, has an average particle diameter of about 6 µm [14]. 

We previously reported that the addition of phosphate compounds such as H**_3_**PO**_4_**, Na**_3_**PO**_4_**, Na**_2_**HPO**_4_** or NaH**_2_**PO**_4_** to BiSCaO or BiSCa(OH)_2_ suspensions results in the formation of dispersion or colloidal dispersion [14]. The present study describes a method for preparing BiSCaO and BiSCa(OH)_2_ colloidal dispersions by mixing with PP or TP as flocculent agents. Two layers quickly form: a supernatant and a flocculants/precipitates composed of polymeric colloidal calcium phosphate (Table 2). The present study showed that those BiSCaO and BiSCa(OH)_2_ colloidal dispersions significantly reduced the number of microorganisms by flocculation/precipitation and the strong microbicidal effect by their hydroxide alkalinity. The TC and CF cell counts in supernatants of the BiSCaO and BiSCa(OH)_2_ colloidal dispersions under strongly alkaline condition were much lower compared with samples under weak alkaline and neutral condition, and the microbicial effect was more pronounced with BiSCaO colloidal dispersions than with BiSCa(OH)_2_ colloidal dispersions at each pH (pH 5–12) (Figure 5). 

On the other hand, the BiSCaO colloidal dispersion containing PP generated flocculants/precipitates adsorbed greater amounts of compounds/particulates such as trypan blue, albumin, chondroitin sulfate, heparin, NAC-HCPS and LMWH/P NPs compared to BiSCa(OH)**_2_** colloidal dispersions. The zeta potentials of BiSCaO and BiSCa(OH)_2_ colloidal dispersions under highly alkaline conditions (pH 11.5–12.2) were positive but were weakly negative at weakly alkaline and neutral conditions (pH 7–10.6) (Table 2). These results indicate that lattice colloidal materials in both BiSCaO and BiSCa(OH)_2_ colloidal dispersions under highly alkaline conditions are positive and could adsorb negatively charged organic compounds as well as microbials. NAC-HCPS [19,26] and LMWH/P NPs in water were present in the suspensions as nanoparticles with an average diameter of 220–230 nm and a zeta-charge of about -30 mV, and 50–200 nm in diameter and a zeta charge of −25 to −30 mV [20], respectively. The zeta charges of both NAC-HCPS and LMWH/P NPs in water are constantly negative at alkaline conditions (pH = 12). Thus, BiSCaO and BiSCa(OH)_2_ colloidal dispersions under highly alkaline conditions effectively separate NAC-HCPS and LMWH/P NPs, more efficiently than water-soluble chondroitin sulfate and heparin. Furthermore, CaO particles remaining in suspension were surrounded by cotton/fibrous lattice colloidal calcium phosphate in BiSCaO colloidal dispersions under strongly alkaline conditions, reinforcing flocculation/precipitation by adsorbing various compounds as well as having microbicidal activity. Currently, we have started study on alkaline flocculation/precipitation for organophosphorus poison and dioxin-like compounds as well as organic matter from bacteria such as endotoxin.

CaO hydration generates a strong base and is the primary mechanism for the disinfection and decontamination activities of BiSCaO colloidal dispersions. The CaO content of BiSCaO is much higher (99.6%) than that of BiSCa(OH)_2_, suggesting that BisCaO colloidal dispersion shows higher microbicidal activities than BiSCa(OH)_2_. Indeed, the pH of 0.2 wt.% BiSCaO, and BiSCa(OH)_2_ containing 0.15 wt.% PP colloidal dispersion is 12.65 and 12.21, respectively (Table 2). However, the bactericidal and flocculation/precipitation activities of BiSCaO colloidal dispersions are higher than those of BiSCa(OH)_2_ colloidal dispersions at the same pH (pH 7–12.2). This suggests that alkalinity alone is not responsible for the flocculation/precipitation and microbicidal properties of BiSCaO. We previously suggested that BiSCaO can reduce CaO. Therefore, another possibility for the high antimicrobial activity of BiSCaO is that the OH^−^ concentration of the thin water layer formed around BiSCaO particles might be higher than that of the bulk solvent [11,14]. Furthermore, active radical species generated from CaO may also contribute to stronger antimicrobial activity [3,9], as supported by a multi-parameter flow cytometry study conducted by Hewitt et al. [27]. The active radical species produced by MgO or CaO is currently poorly understood. Krishnamoorthy et al. [28] investigated the antibacterial activity of MgO, which, like CaO, is an alkaline earth metal oxide. They suggested that the antibacterial activity of MgO relies on the presence of defects or oxygen vacancies at the surface of the particles. Since MgO is easily hydrated and forms a surface layer of Mg(OH)_2_, it readily establishes surface-bound electron-hole pairs that can decompose into a surface-trapped electron and a localized hole state [29,30]. Further studies are required to understand the mechanism by which CaO exerts its antimicrobial and decontamination effects.

## 5. Conclusions

BiSCaO and BiSCa(OH)_2_ are poorly water-soluble under strongly alkaline conditions (pH > 11.5) and generate precipitations. In this study, 0.2 wt.% BiSCaO and BiSCa(OH)_2_ colloidal dispersions were prepared by mixing with 0.15 wt.% PP or TP. The resulting BiSCaO colloidal dispersions (pH 12.2) exhibited the highest microbicidal effects and ability to flocculate/precipitate trypan blue, albumin, chondroitin sulfate, heparin, NAC-HCPS, and LMWH/P NPs as negatively charged organic compounds or nanoparticles in comparison to BiSCa(OH)_2_ colloidal dispersions.

## Figures and Tables

**Table 1:** The effects of adding various amounts of PP or TP to 0.2 wt.% BiSCaO or BiSCa(OH)_2_ suspensions.

**BiSCaO**	**pH** **(Conc. of PP and TP)**	**12.2** **(0.05 %)**	**12.2** **(0.1 %)**	**12.2** **(0.15 %)**	**12.2** **(0.2 %)**	**12.2** **(0.25 %)**	**12.2** **(0.30 %)**
	Flocculants (vol.%)	10	15	15	15	15	20
	Appearance	Dispersion with flocculants/ precipitates	Easy dispersible flocculants/ precipitates	Easy dispersible flocculants/ precipitates	Easy dispersible flocculants/ precipitates	Easy dispersible flocculants/ precipitates	Easy dispersible flocculants/ precipitates
**BiSCa** **(OH)_2_**	**pH** **(Conc. Of PP and TP)**	**12.23** **(0.05 %)**	**12.22** **(0.1 %)**	**12.21** **(0.15 %)**	**12.2** **(0.2 %)**	**12.2** **(0.25 %)**	**12.2** **(0.30 %)**
	Flocculants (vol.%)	10	15	15	15	20	25
	Appearance	Dispersion with flocculants/ precipitates	Easy dispersible flocculants/ precipitates	Easy dispersible flocculants/ precipitates	Easy dispersible flocculants/ precipitates	Easy dispersible flocculants/ precipitates	Easy dispersible flocculants/ precipitates

**Table 2:** Effect of pH on generating flocculants/precipitates in 0.2 wt.% BiSCaO and BiSCa(OH)_2_ colloidal dispersions containing 0.15 wt.% PP or TP.

**BiSCaO**	**pH**	**12.65**	**12.2**	**11.8**	**10.0**	**7.7**	**4.7**
	Flocculants (vol.%)	7	15	15	10	10	0
	Appearance	Dispersion with flocculants/ precipitates	Easy dispersible flocculants/ precipitates	Easy dispersible flocculants/ precipitates	Easy dispersible flocculants/ precipitates	Easy dispersible flocculants/ precipitates	Soluble
	Zeta potential (mV)	+5.1 ± 1.9	+14.7 ± 1.6	+11.2 ± 1.8	−1.4 ± 0.2	−5.6 ± 0.8	Not measured
**BiSCa** **(OH)_2_**	**pH**	**12.23**	**11.5**	**10.6**	**9.6**	**7.2**	**5.6**
	Flocculants (vol.%)	15	15	15	10	10	0
	Appearance	Easy dispersible flocculants/ precipitates	Easy dispersible flocculants/ precipitates	Easy dispersible flocculants/ precipitates	Easy dispersible flocculants/ precipitates	Easy dispersible flocculants/ precipitates	Dissolution
	Zeta potential (mV)	+16.2 ± 2.0	+10.8 ± 1.9	−2.1 ± 0.6	−4.3 ± 0.5	−4.5 ± 0.6	Not measured

**Table 3:** Removal of trypan blue using BiSCaO and BiSCa(OH)_2_ colloidal dispersions.

**BiSCaO**	**pH** **(Conc. of PP and TP)**	12.23 (0.05%)	12.23 (0.1%)	12.22 (0.15%)	12.22 (0.2%)	12.21 (0.25%)	12.2 (0.30%)
	**OD_650_ (PP)**	0.25 ± 0.03	0.10 ± 0.02	0.03 ± 0.01	0.14 ± 0.02	0.22 ± 0.05	0.44 ± 0.11
	**OD_650_ (TP)**	0.2 ± 0.03	0.09 ± 0.02	0.05 ± 0.02	0.12 ± 0.03	0.2 ± 0.03	0.42 ± 0.10
**BiSCaO with 0.15 wt.% PP and TP**	**pH**	12.65	12.2	11.8	10.0	7.7	4.7
	**OD_650_ (PP)**	0.66 ± 0.22	0.03 ± 0.01	0.05 ± 0.02	0.38 ± 0.08	0.65 ± 0.11	0.92 ± 0.21
	**OD_650_ (TP)**	0.65 ± 0.18	0.11 ± 0.02	0.12 ± 0.02	0.60 ± 0.11	0.78 ± 0.16	1.19 ± 0.18
**BiSCa(OH)_2_**	**pH** **(Conc. of PP and TP)**	12.2 (0.05%)	12.23 (0.1%)	12.22 (0.15%)	12.22 (0.2%)	12.21 (0.25%)	12.2 (0.30%)
	**OD_650_ (PP)**	0.28 ± 0.03	0.16 ± 0.02	0.15 ± 0.02	0.18 ± 0.02	0.22 ± 0.05	1.24 ± 0.21
	**OD_650_ (TP)**	0.3 ± 0.06	0.19 ± 0.04	0.17 ± 0.04	0.18 ± 0.03	0.33 ± 0.06	1.29 ± 0.2
**BiSCa(OH)_2_ with 0.15 wt.% PP and TP**	**pH**	12.2	11.5	10.6	9.6	7.2	5.6
	**OD_650_ (PP)**	0.15 ± 0.02	0.2 ± 0.05	0.43 ± 0.07	0.77 ± 0.14	0.79 ± 0.16	1.42 ± 0.33
	**OD_650_ (TP)**	0.2 ± 0.04	0.23 ± 0.06	0.47 ± 0.08	0.84 ± 0.2	0.88 ± 0.22	1.59 ± 0.38

The OD_650_ of trypan blue at pH 4.7–12.65 without BiSCaO or BiSCa(OH)_2_ was 1.6 ± 0.02.

**Table 4:** Removal of albumin using BiSCaO and BiSCa(OH)_2_ colloidal dispersions.

BiSCaO	pH (Conc. of PP and TP)	12.2 (0.05%)	12.19 (0.1%)	12.2 (0.15%)	12.19 (0.2%)	12.2 (0.25%)	12.2 (0.30%)
	OD_562_ (PP)	0.45 ± 0.12	0.22 ± 0.06	0.11 ± 0.01	0.18 ± 0.02	0.32 ± 0.08	0.74 ± 0.16
	OD_562_ (TP)	0.52 ± 0.14	0.29 ± 0.02	0.22 ± 0.04	0.24 ± 0.05	0.43 ± 0.12	0.84 ± 0.20
BiSCaO with 0.15 wt.% PP and TP	pH	12.65	12.2	11.8	10.0	7.7	4.7
	OD_562_ (PP)	0.73 ± 0.21	0.11 ± 0.01	0.14 ± 0.02	0.36 ± 0.08	0.75 ± 0.11	0.96 ± 0.21
	OD_562_ (TP)	0.75 ± 0.22	0.22 ± 0.04	0.18 ± 0.03	0.51 ± 0.11	0.88 ± 0.16	1.19 ± 0.18
BiSCa(OH)_2_	pH (Conc. of PP and TP)	12.2 (0.05%)	12.21 (0.1%)	12.21 (0.15%)	12.19 (0.2%)	12.21 (0.25%)	12.2 (0.30%)
	OD_562_ (PP)	0.51 ± 0.11	0.36 ± 0.08	0.21 ± 0.04	0.42 ± 0.15	0.64 ± 0.19	1.12 ± 0.21
	OD_562_ (TP)	0.63 ± 0.16	0.48 ± 0.09	0.33 ± 0.06	0.48 ± 0.16	0.73 ± 0.21	1.29 ± 0.31
BiSCa(OH)_2_ with 0.15 wt.% PP and TP	pH	12.21	11.2	10.4	9.6	7.2	4.6
	OD_562_ (PP)	0.21 ± 0.04	0.27 ± 0.07	0.48 ± 0.08	0.67 ± 0.18	0.87 ± 0.19	1.34 ± 0.36
	OD_562_ (TP)	0.33 ± 0.06	0.38 ± 0.09	0.51 ± 0.11	0.64 ± 0.21	0.92 ± 0.22	1.43 ± 0.39

The OD_562_ of albumin at pH = 7.5 without BiSCaO or BiSCa(OH)_2_ was 1.47 ± 0.02.

**Table 5:** Removal of chondroitin sulfate, heparin, NAC-HCPS and LMWH/P NPs using BiSCaO and BiSCa(OH)_2_ colloidal dispersions.

**Chondroitin sulfate**	**BiSCaO** **(PP; 0.15 wt.%)**	**pH**	12.63	12.1	11.5	10.2	7.8	5.6
		**OD_656_ (PP)**	0.71 ± 0.25	0.17 ± 0.04	0.32 ± 0.12	0.66 ± 0.19	0.89 ± 0.21	1.44 ± 0.31
	**BiSCa(OH)_2_** **(PP; 0.15 wt.%)**	**pH**	12.2	11.4	10.5	9.8	7.5	5.5
		**OD_656_ (PP)**	0.23 ± 0.07	0.44 ± 0.11	0.63 ± 0.12	0.77 ± 0.21	0.95 ± 0.27	1.42 ± 0.33
**Heparin**	**BiSCaO** **(PP; 0.15 wt.%)**	**pH**	12.64	12.2	11.7	10.1	7.6	5.7
		**OD_656_ (PP)**	0.77 ± 0.22	0.42 ± 0.12	0.58 ± 0.14	0.91 ± 0.26	0.13 ± 0.31	1.52 ± 0.33
	**BiSCa(OH)_2_** **(PP; 0.15 wt.%)**	**pH**	12.2	11.6	10.6	9.5	7.3	5.6
		**OD_656_ (PP)**	0.56 ± 0.18	0.72 ± 0.25	1.14 ± 0.27	1.27 ± 0.31	1.45 ± 0.41	1.55 ± 0.36
**NAC-HCPS**	**BiSCaO** **(PP; 0.15 wt.%)**	**pH**	12.65	12.2	11.5	10.0	7.8	5.7
		**OD_656_ (PP)**	0.65 ± 0.2	0.03 ± 0.01	0.16 ± 0.04	0.42 ± 0.08	0.76 ± 0.17	1.22 ± 0.32
	**BiSCa(OH)_2_** **(PP; 0.15 wt.%)**	**pH**	12.2	11.6	10.4	9.9	7.3	5.3
		**OD_656_ (PP)**	0.26 ± 0.07	0.35 ± 0.12	0.57 ± 0.18	0.72 ± 0.24	0.88 ± 0.16	1.45 ± 0.32
**LMWH/P NPs**	**BiSCaO** **(PP; 0.15 wt.%)**	**pH**	12.65	12.2	11.5	10.0	7.8	5.7
		**OD_656_ (PP)**	0.43 ± 0.1	0.04 ± 0.01	0.18 ± 0.0.4	0.46 ± 0.09	0.79 ± 0.24	1.28 ± 0.33
	**BiSCa(OH)_2_** **(PP; 0.15 wt.%)**	**pH**	12.2	11.6	10.4	9.9	7.3	5.3
		**OD_656_ (PP)**	0.23 ± 0.07	0.43 ± 0.14	0.82 ± 0.29	0.97 ± 0.28	1.29 ± 0.36	1.49 ± 0.33

The OD_456_ of chondroitin sulfate, heparin, and NAC-HCPS and LMWH/P NPs at pH = 7.6 without BiSCaO and BiSCa(OH)_2_ was 1.45 ± 0.02, 1.55 ± 0.02, 1.46 ± 0.02, and 1.51 ± 0.02, respectively.
